# Kynurenic acid protects against ischemia/reperfusion injury by modulating apoptosis in cardiomyocytes

**DOI:** 10.1007/s10495-024-02004-w

**Published:** 2024-08-17

**Authors:** Renáta Gáspár, Dóra Nógrádi-Halmi, Virág Demján, Petra Diószegi, Nóra Igaz, Anna Vincze, Márton Pipicz, Mónika Kiricsi, László Vécsei, Tamás Csont

**Affiliations:** 1 Metabolic Diseases and Cell Signaling Research Group, Department of Biochemistry, Albert Szent-Györgyi Medical School, University of Szeged, Dóm Tér 9, 6720 Szeged, Hungary; 2 Interdisciplinary Centre of Excellence, University of Szeged, Szeged, Hungary; 3 Department of Biochemistry and Molecular Biology, Faculty of Science and Informatics, University of Szeged, Szeged, Hungary; 4 Department of Neurology, Albert Szent-Györgyi Health Centre, University of Szeged, Szeged, Hungary; 5 HUN-REN-SZTE-Neuroscience Research Group, Szeged, Hungary

**Keywords:** Ischemia/reperfusion injury, Apoptosis, Kynurenic acid, Trp metabolites

## Abstract

Acute myocardial infarction, often associated with ischemia/reperfusion injury (I/R), is a leading cause of death worldwide. Although the endogenous tryptophan metabolite kynurenic acid (KYNA) has been shown to exert protection against I/R injury, its mechanism of action at the cellular and molecular level is not well understood yet. Therefore, we examined the potential involvement of antiapoptotic mechanisms, as well as N-methyl-D-aspartate (NMDA) receptor modulation in the protective effect of KYNA in cardiac cells exposed to simulated I/R (SI/R). KYNA was shown to attenuate cell death induced by SI/R dose-dependently in H9c2 cells or primary rat cardiomyocytes. Analysis of morphological and molecular markers of apoptosis (i.e., membrane blebbing, apoptotic nuclear morphology, DNA double-strand breaks, activation of caspases) revealed considerably increased apoptotic activity in cardiac cells undergoing SI/R. The investigated apoptotic markers were substantially improved by treatment with the cytoprotective dose of KYNA. Although cardiac cells were shown to express NMDA receptors, another NMDA antagonist structurally different from KYNA was unable to protect against SI/R-induced cell death. Our findings provide evidence that the protective effect of KYNA against SI/R-induced cardiac cell injury involves antiapoptotic mechanisms, that seem to evoke independently of NMDA receptor signaling.

**Supplementary Information:**

The online version contains supplementary material available at 10.1007/s10495-024-02004-w.

## Introduction

Pathological conditions associated with ischemia/reperfusion (I/R), including acute myocardial infarction (AMI), are among the leading causes of death globally. Myocardial I/R injury occurs when ischemia exceeds a critical threshold and overwhelms the cardiac cellular repair mechanisms designed to maintain physiological function and homeostasis, consequently resulting in irreversible myocardial cell damage and cell death [1]. The I/R-induced cellular damage is a multifaceted mechanism, involving induction of oxidative stress, mitochondrial dysfunction, dysregulation of survival processes, as well as activation of various cell death pathways [2, 3]. The I/R-induced cellular demise occurs as a combination of both unregulated (i.e., necrosis) and regulated (i.e., apoptosis) cell death mechanisms. A considerable amount of literature has been published on the pivotal role of apoptosis in cardiac I/R-injury, suggesting that it contributes to both short- and long-term loss of cardiomyocytes in the tissue adjacent to the infarcted myocardium. Apoptosis determines the extent a myocardial injury and it has been reported to participate in the development of ventricular remodeling and heart failure following AMI, and is considered as a major determinant of AMI-related morbidity and mortality [4–8]. Although for this reason pharmacological amelioration of myocardial apoptosis represents a promising target in the development of novel therapeutic strategies, so far there is no clinically applicable drug available to inhibit myocardial apoptosis in AMI patients [9].

Kynurenic acid (KYNA) is a biologically active natural compound produced during the catabolism of tryptophan through the kynurenine pathway, which received extensive scientific attention over the past decades, mainly in the field of experimental neurology [10, 11]. Multiple studies suggest that KYNA exerts protection against ischemia/anoxia-induced neuronal injury [12–14], most likely through the modulation of receptor-mediated signaling pathways by acting on various receptors including N-methyl-D-aspartate receptor (NMDA), aryl hydrocarbon (AhR) or G protein-coupled receptor 35 (GPR35) receptors (Supplement. Table 1.) [15]. Stimulation of NMDA receptors has been shown to trigger neuronal apoptosis [16, 17], and has been implicated in ischemic stroke-induced neuronal death [18] as well. Even though the role of KYNA in both physiological and pathological processes has been thoroughly studied in the brain, fewer investigations have been carried out on the function and actions of KYNA in the peripheral tissues. Previous research indicated anti-inflammatory [19], analgesic [20], antioxidative [21] and hepatoprotective properties of KYNA [22], which support its potential beneficial role outside of the nervous system. Interestingly, although some studies suggested that increased serum level of KYNA predicts higher risk for AMI [23–25]; it also has been shown that physical exercise increases the concentration of KYNA in parallel with the reduction of kynurenine-mediated toxicity, which in turn regulates energy expenditure and improves the tolerance of the heart against ischemic conditions [26, 27]. In addition, Olenchock et al*.* revealed that elevation of liver-derived KYNA in the serum is essential for the I/R injury-limiting effect of remote ischemic conditioning, and showed that the administration of KYNA significantly decreased the infarct size in mice [28]. Nevertheless, the thorough mechanism of action of KYNA as a potential cardioprotective agent has hardly been investigated. An interesting research conducted parallel with our current study proposed that binding of KYNA by GPR35 receptors initiates a sequence of molecular steps which lead to dimerization of ATP synthase in the mitochondria, thereby promoting energy conservation [29]. In the present study we aimed to further investigate the potential molecular and cellular mechanisms contributing to the cardiocytoprotective effect of KYNA. As apoptotic cell death plays a fundamental role in I/R-induced cardiac tissue damage, here we aimed to analyze if KYNA administration counteracts the I/R-triggered proapoptotic signaling. Furthermore, as activation of NMDA-receptors has been implicated in I/R-induced neuronal apoptosis, we examined whether the cardiocytoprotective feature of KYNA involves the modulation of NMDA receptor signaling.

## Materials and methods

### H9c2 cell culture

H9c2 rat cardiomyoblasts were obtained from ATCC (ATCC; St. Louis, MO, USA, RRID: CVCL_0286) and cultured in Dulbecco’s modified Eagle’s medium (DMEM; Biosera, Cat#LM D1108) supplemented with 10% v/v fetal bovine serum (FBS; Biosera, Cholet, France, Cat#FB1090), 200 nM glutamine (Sigma-Aldrich, St. Louis, MO, USA, Cat#G7513) and 1% v/v antibiotic/antimycotic cocktail (Sigma-Aldrich, Cat#A5955). Cells were cultured in 25 cm^2^ and 75 cm^2^ tissue culture flasks and were subcultured each time they reached 70–80% of confluence. Cells from passage P15-20 were seeded at a density of 4 × 10^3^ cells/well into 96-well plates for viability, lactate dehydrogenase (LDH) and caspase 3/7 activity measurements, 5 × 10^3^ cells/well into 96-well plates for 5-Bromo-2´-Deoxyuridine (BrdU) assay and 5 × 10^4^ cells/well into 24-well plates for wound-healing assay. For immunocytochemistry assays, cells were seeded into 24-well plates containing coverslips at a density of 2 × 10^4^ cells/well. To prepare western blot samples, cells were grown in 75 cm^2^ tissue culture flasks. Cells were subjected to the different experimental protocols two days after cell seeding.

### Primary cardiomyocyte cultures

Primary cultures of neonatal rat cardiomyocytes were isolated from 1 to 3 days old Wistar rats (Charles River Laboratories, Wilmington, Massachusetts, USA) according to a previously published protocol [30]. Cells were seeded at a density of 1.5 × 10^4^ cells/well into 96-well plates for the measurement of viability, 5 × 10^4^ cells/well into 24-well plates containing coverslips for immunocytochemistry or 3.5 × 10^5^ cells/well into 6-well plates for the preparation of western blot samples. Cultures were grown under DMEM containing 10% v/v FBS, 1% v/v glutamine and 1% v/v antibiotic/antimycotic solution for 24 h in a standard CO_2_ incubator (95% air, 5% CO_2_). One day later, growth medium was replaced by DMEM supplemented with 1% v/v FBS. Experiments were carried out 3 days after plating.

### Experimental design and SI/R

To determine the potential protective treatment concentration of KYNA, a 10 mM stock solution was prepared by dissolving KYNA powder (Sigma Aldrich, Cat#K3375) in 0.025 M NaOH and setting its pH to 7.38–7.42. In a preliminary experiment, KYNA was tested in a 16–256 µM concentration range to assess the dose-dependent reduction of cell death upon KYNA treatment. For subsequent investigations on KYNA-induced molecular and cellular mechanisms, 64 µM concentration of KYNA was applied. To investigate the potential cytoprotective effect of KYNA, H9c2 cardiomyoblasts were subjected to 6 h of simulated ischemia followed by 2 h of simulated reperfusion, carried out as described previously [30]. KYNA treatment was maintained during the entire SI/R protocol. To prove the effectiveness of SI/R as a stress factor, a control group of cells was kept under normoxic conditions (i.e., under normoxic solution/media in a standard CO_2_ incubator) throughout the protocol [19]. Viability assays and LDH activity measurements were performed to analyze the effect of KYNA on the degree of SI/R-induced cell death. To assess whether KYNA modulates programmed cell death in this setting, morphological and molecular hallmarks of apoptosis were assessed using light microscopy, immunocytochemistry, and western blot.

To investigate the potential involvement of NMDA receptors in the pathophysiology of SI/R as well as in the KYNA-induced molecular events, H9c2 cardiomyoblasts exposed to SI/R were treated with an NMDA receptor antagonist, MK-801 hydrogen maleate (0.47–120 µM, Sigma-Aldrich, Cat#M107). In separate experiments, cells subjected to SI/R were treated with the NMDA receptor agonist N-Methyl-D-aspartic acid (NMDA, Sigma-Aldrich, Cat#M3262) or with the combination of NMDA and MK-801 or NMDA and KYNA.

To confirm the KYNA-derived cardiocytoprotection on primary cells, three-day old primary cardiomyocyte cultures were subjected to 4 h of simulated ischemia followed by 2 h of simulated reperfusion as described previously [30]. After the protocol, viability assays, western blot and immunocytochemistry were performed.

### Camptothecin-induced apoptotic cell death model

To confirm that the cytoprotective effect of KYNA involves antiapoptotic mechanisms, we assessed whether the administration of KYNA improves the viability of cells exposed to treatment with an apoptosis inducer. To induce programmed cell death, Camptothecin (Cell Signaling Technology, Danvers, MA, USA, Cat#13637) was applied at a concentration of 10 µM, dissolved in DMEM supplemented with 1% v/v FBS. H9c2 cells received a 30 min long pre-treatment with 8–128 µM KYNA or its vehicle before exposure to Camptothecin for 24 h. The corresponding KYNA/vehicle treatments were maintained during the 24 h of Camptothecin treatment. At the end, 3-(4,5-dimethylthiazol-2-yl)-2,5-diphenyltetrazolium bromide (MTT) assay was performed to measure cell viability.

### Viability assays

Calcein (Thermo Fisher Scientific, Waltham, Massachusetts, USA, Cat#C3100MP) staining was applied after SI/R to determine cell viability according to a previously published protocol using a fluorescent plate reader (BMG ClarioStar Plus/Fluostar Optima, BMG Labtech, Ortenberg, Germany) [30, 31]. Viability measurements were repeated at least 3 times with at least 7–14 biological replicates. Cell death was determined as percentage of SI/R-induced cell death (the difference between the average viability detected in the normoxic group and that of the vehicle treated hypoxic groups). MTT assay was used to determine cell viability in cells exposed to Camptothecin treatment. At the end of the experimental protocol, supernatant was replaced with 1% v/v FBS-containing DMEM supplemented with 0.5 mg/mL MTT (Sigma-Aldrich, Cat#M2128) (1 h, 37 °C). Formazan crystals were dissolved thoroughly in dimethyl-sulfoxide (Serva, Heidelberg, Germany, Cat#67-68-5), and optical density was measured at 570 nm using a plate reader (BMG ClarioStar Plus/Fluostar Optima).

### Measurement of cellular superoxide levels

To investigate the effect of KYNA on SI/R-induced cellular superoxide production, dihydro-ethidium (DHE) staining was performed as described previously [30]. During simulated ischemia, cells were covered with hypoxic solution supplemented with various concentrations of KYNA or its vehicle. During the 2 h of simulated reperfusion, cells were covered with 1% FBS-containing media supplemented with KYNA/vehicle and 10 µM DHE. After washing, fluorescence intensity was measured at 530/620 nm followed by detection of cell viability using Calcein assay. The fluorescence intensity of DHE was normalized to cell viability. The level of oxidative stress was expressed as percentage of oxidative stress in the normoxic or vehicle-treated control groups.

### Measurement of lactate dehydrogenase (LDH) activity

To confirm the effect of KYNA against SI/R-induced cardiomyocyte cell death, LDH enzyme activity was measured after SI/R from the supernatants of H9c2 cells using LDH activity assay kit according to manufacturer’s instructions (Cat#416465, Diagnosticum Inc., Budapest, Hungary).

### Investigation of apoptotic membrane blebbing

To investigate whether KYNA affects apoptotic cell morphology, apoptotic membrane blebbing was examined after SI/R with or without KYNA treatment. At the end of the simulated reperfusion, cells were washed and collected from 6-well plates using 0.25% trypsin–EDTA solution (Corning, NY, USA, Cat#25–053-Cl; 5 min, 37˚C) followed by centrifugation (5 min, room temperature (RT), 400 g). After the removal of supernatants, cell pellets were resuspended in DMEM containing 10% v/v FBS. For visualization, 15 µl of samples were placed onto slides, and 6–10 fields were captured using Leica DMi1 inverted light microscope (Leica Mycrosystems, Wetzlar, Germany). The experiments were repeated three times. An observer blinded to the experimental conditions counted all cells and scored those with obvious membrane protrusions as blebbing cells using the Image J Software (version 1.8.0_112, National Institutes of Health, Bethesda, MD, USA) with cell counter plugin. Cells were classified into the 5 stages of membrane blebbing considering the following criteria: (i) normal, healthy cells with rounded, intact, sharp cell contour belonged to Stage 1; (ii) cells showing unchanged shape and size, but altered membrane refraction were sorted into Stage 2; (iii) cells exhibiting cell-surface blebbing characterized by reversible formation of peripheral circular bulges belonged to Stage 3; (iv) cells showing dynamic blebs (i.e., a sign of late, irreversible apoptotic phase) were sorted into Stage 4; while (v) cells showing drastically changed shape or signs of final fragmentation were categorized into Stage 5. Number of cells in each stage was expressed as percentage of total cell number [32–36].

### Assessment of alterations in nuclear morphology

To determine the ratio of cells showing apoptotic nuclear morphology, cells were seeded onto glass coverslips at a density of 2 × 10^4^ cells/well and were grown in 24-well plates. After two days, cells underwent SI/R with or without KYNA treatment. Normoxic samples were prepared as controls. After the protocol, cells were fixed in 4% paraformaldehyde (PFA; Alfa Aesar, Haverhill, MA, USA, Cat#30525-89-4) (20 min, RT). Permeabilization was performed using 0.3% Triton X-100-containing phosphate buffered saline (PBS) (20 min, RT), followed by blocking in 5% bovine serum albumin-containing PBS (30 min, RT). Cell nuclei were visualized using 4,6-diamidino-2-phenylindole (DAPI; Abcam, Cambridge, United Kingdom, Cat#ab228549) staining (1:10,000, 10 min, RT). Coverslips were mounted onto slides using Mounting Medium, followed by the visualization of cells with Nikon Eclipse Ti E microscope (Nikon Instruments Inc., NY, USA). Images were captured of at least 5 randomly selected fields of view using the same exposition time for all samples. Image J software with cell counter plugin was used for image analysis. The number of cell nuclei showing definite condensation, micronucleoli formation or fragmentation was quantified and normalized to the total number of cell nuclei on each field of view to express the ratio of apoptotic nuclei in each group.

### Immunocytochemistry

For immunocytochemical detection of apoptotic markers (i.e., caspase-3, caspase-8, DNA double-strand breaks (phosphorylated histone 2A variant X (γ-H2AX)), H9c2 cells and/or primary neonatal cardiomyocytes were seeded onto glass coverslips at a density of 2 × 10^4^ cells/well or 5 × 10^4^ cells/well, respectively, and were grown for two days. Cells subjected to SI/R were treated with 64 µM (H9c2 cells) or 128 µM (primary cardiomyocytes) KYNA or its vehicle. As controls of the experiment, normoxic samples were prepared. At the end of the simulated reperfusion, cells were fixed in 4% PFA (20 min, RT). Cells then were permeabilized using 0.3% Triton X-100-containing PBS (20 min, RT), and blocked in 5% bovine serum albumin (BSA, VWR, Radnor, PA, USA, Cat#9048-46-8)-containing PBS (30 min, RT). Cells were then incubated with primary antibodies against pro- and cleaved forms of caspase-3 (1:100, overnight, 4 °C), caspase-8 (1:150, overnight, 4 °C) and γ-H2AX (1:300, overnight, 4 °C) in a humidified chamber. Samples were then washed with PBS supplemented with 0.1% Tween20, followed by incubation with Alexa-488/Alexa-647 fluorophore-conjugated Goat Anti-Rabbit or Goat Anti-Mouse secondary antibodies (1:200–300, 40 min, RT). All applied antibodies are listed in Supplementary Table 2. Cell nuclei were stained with DAPI (1:10,000, 10 min, RT). Coverslips were covered with Mounting Medium, and cells were visualized with a Nikon Eclipse Ti-E microscope (Nikon Instruments Inc., NY, USA) using the same exposition time for all samples. Images were captured of at least 5 randomly selected fields of view per sample. Raw images were processed by observers blinded to the experimental conditions using Image J software. For immunocytochemical investigations involving fluorescence intensity measurement, images were converted into 8-bit greyscale file format. For the detection of fluorescence intensity, mean grey value was measured and normalized to cell count. Images with poor image quality or imperfect staining were excluded from the evaluation.

### Caspase-3/7 activity measurement

To further corroborate whether KYNA influences the SI/R-induced alterations of proapoptotic pathways, H9c2 cells were subjected to SI/R with or without KYNA treatment, followed by caspase-3/7 activity measurement using Caspase-Glo 3/7 Assay (Promega, Madison, Wisconsin, USA; Cat#G8090) according to the manufacturer’s instructions.

### Western blot analysis

To harvest samples for western blotting, H9c2 cardiomyoblasts and neonatal cardiomyocytes were grown in 75 cm^2^ tissue culture flasks or 6-well plates, respectively, and subjected to SI/R while they were treated with 64/128 µM KYNA or its vehicle. Sample preparation and measurement of protein concentration was performed as described previously [30]. 25 µg protein was loaded onto and was separated in 10, 12 or 15% polyacrylamide gels. Proteins were blotted onto 0.2 µm pore-size nitrocellulose membranes (35 V, 1.5 h, RT in case of Bcl-2 associated X (BAX), B-cell lymphoma extra-large (Bcl-XL), B-cell lymphoma 2 (Bcl-2) and receptor-interacting serine/threonine-protein kinase 1 (RIPK1)) or onto 0.45 µm pore-size PVDF membranes (35 V, 45 min, RT in case of γ-H2AX or 35 V, 60 min, RT in case of caspase-3 and -7). Membranes were cut horizontally with respect to the molecular weights of the target proteins followed by blocking in 5% v/v BSA (1 h, RT). Membranes were then incubated with specific primary antibodies against γ-H2AX, BAX, Bcl-XL, Bcl-2, caspase-3, caspase-7, RIPK1 (1:1000), α-tubulin (1:2000), β-actin (1:1500) and GAPDH (1:10,000) dissolved 1% v/v BSA (overnight, RT or 4 °C), followed by incubation with HRP-conjugated goat anti-rabbit secondary antibody (120 min, RT). Primary and secondary antibodies used for western blotting are listed in Supplementary Table 2. Membranes were detected using LumiGlo 20X reagent (Cell Signaling Technology, Cat#7003) and exposure to X-ray films. All films were scanned (8-bit, 400 dpi) and the density of each protein band was quantified using Quantity One software 4.4.0.36 (Bio-Rad company, Hercules, California) [37–39].

### Statistical analysis

All data were expressed as mean + standard error of the mean (S.E.M.) except stated otherwise. The normality of sample distribution was confirmed using Shapiro-Wilks normality test. In case of normal distribution, the comparisons between two groups were determined by Student’s t test using Prism 8.0 software (GraphPad Software, GraphPad Software Ldt., California, USA). Comparisons involving more than two groups were assessed by one-way ANOVA with Tukey’s multiple comparisons test as post hoc analysis. In case of non-normal sample distribution, Mann–Whitney, and Kruskal–Wallis tests (Dunn post hoc test) were applied according to the number of groups. The following labels were used to indicate significant differences throughout the manuscript: *, #*p* < 0.05 and **, ## *p* < 0.01 in case of t-test or one-way ANOVA, and €, &, $ *p* < 0.05 and €€, &&, $$ *p* < 0.01 in case of non-parametric tests.

## Results

### Kynurenic acid exerts a cytoprotective effect against SI/R injury

**Fig. 1:**
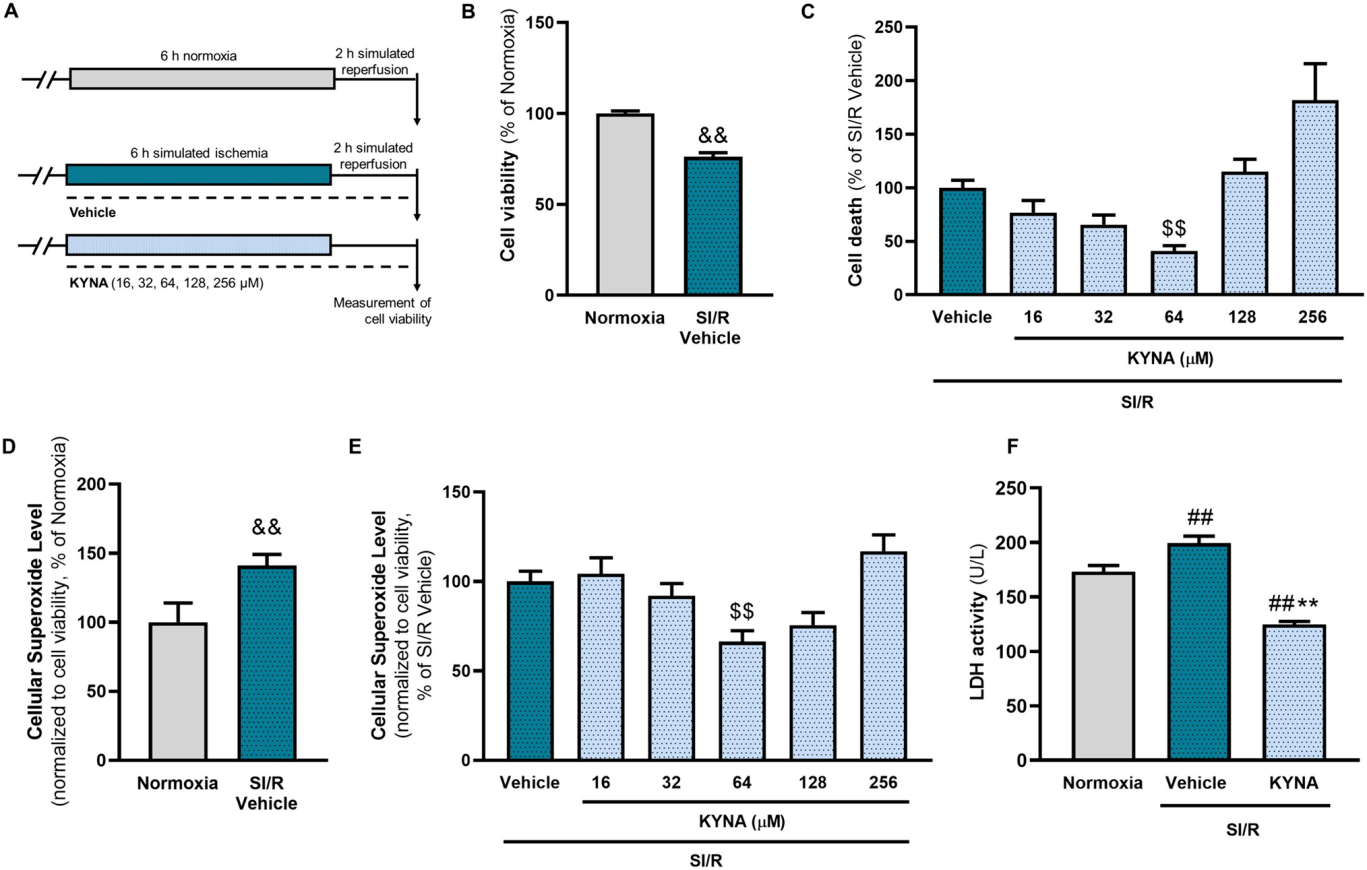
KYNA protects H9c2 cardiomyoblasts against SI/R-induced cell death. **A** H9c2 cardiomyoblasts were subjected to SI/R (i.e., 6 h simulated ischemia followed by 2 h simulated reperfusion), while cells were treated with KYNA or its vehicle. **B** SI/R significantly reduced the viability of H9c2 cells compared to normoxic controls. **C** KYNA treatment protected H9c2 cells against SI/R-induced cell death dose-dependently (*n* = 7–14/experiment (KYNA-treated groups), *n* = 28/experiment (vehicle/normoxic controls); 4 separated experiments). **D** Exposure of cells to SI/R caused a substantial increase in cellular superoxide levels (*n* = 25–50/experiment, 2 independent experiments). **E** 64 µM KYNA treatment was shown to reduce superoxide levels considerably (vehicle-treated control: 21–50/experiment, KYNA-treated groups: 7–14/experiment; 3 independent experiments). **F** The protective effect of 64 µM KYNA was further confirmed via the measurement of LDH release (*n* = 6–7/experiment, 3 independent experiments). Values were normalized to normoxic or vehicle control groups and were expressed as mean ± S.E.M., &&*p* < 0.01 vs. Normoxia, Mann Whitney test; ##*p* < 0.01 vs. Normoxia, **p < 0.01 vs. SI/R + vehicle, one-way ANOVA; $$*p* < 0.01 vs. SI/R + Vehicle, Kruskal–Wallis test

**Fig. 2:**
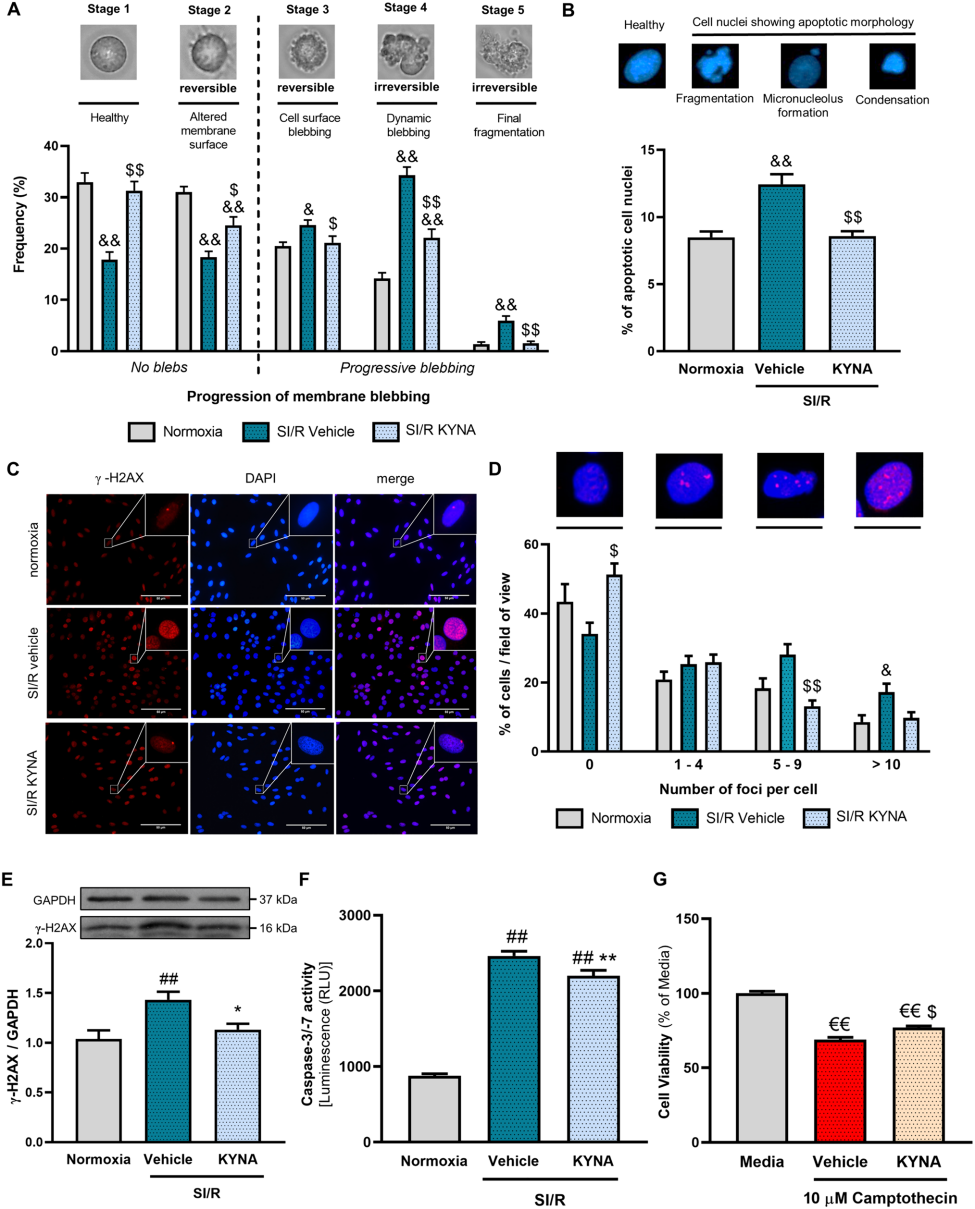
KYNA attenuates the SI/R-induced apoptosis of cardiac cells. **A** Progression of apoptotic membrane blebbing was investigated (*n* = 3–4 sample/experiment, 6–10 randomly selected fields of view/sample, 3 separated experiments) after the SI/R protocol. **B** Visualization of apoptotic nuclear morphology using DAPI staining. Ratio of apoptotic-like nuclei was quantified and normalized to SI/R vehicle group (*n* = 6 from 2 independent experiments, 5–6 randomly selected fields of view/sample). **C–D** The frequency of DNA double-strand break formation was examined by categorizing γH2AX-stained cells accordig to whether they exhibit no DNA double-strand breaks (no γH2AX foci) or show a small (1–4), a moderate (5–9) or a high number (> 9) of DNA breaks per nucleus (*n* = 3–4 from 2 independent experiments, 5–6 randomly selected fields of view/sample; scale bar: 50 µm). **E** γH2AX protein levels were assessed by western blotting (*n* = 6 from 2 independent experiments). **F** Detection of caspase-3/7 activity (*n* = 11–15/experiment, 3 independent experiments). **G** The antiapoptotic activity of KYNA was confirmed on Camptothecin-induced apoptosis: 64 µM of KYNA was applied on H9c2 cardiomyoblasts 30 min prior to and during 24 h treatment with 10 µM Camptothecin (*n* = 14/experiment, 4 separated experiments). Data were expressed as mean ± S.E.M., and were compared to Normoxic, Mediua or Vehicle groups, as appropriate, &&*p* < 0.01 vs. Normoxia, &*p* < 0.05 vs. Normoxia, €€*p* < 0.01 vs. Media, $*p* < 0.05 vs. SI/R + Vehicle, $$*p* < 0.01 vs. SI/R + Vehicle, Kruskal–Wallis test; ##*p* < 0.01 vs. Normoxia, **p < 0.01 vs. SI/R + Vehicle, one-way ANOVA

To test whether KYNA affects cell viability of cardiomyocytes subjected to SI/R, H9c2 cells were treated with various concentrations of KYNA (Fig. 1, Supplement. Figure 1). SI/R significantly decreased the viability of cells compared to that observed in the normoxic controls (Fig. 1B, Supplement. Figure 1B). Interestingly, KYNA treatment showed a dose-dependent effect on SI/R-induced cell death, significantly attenuating it at a concentration of 64 µM (Fig. 1C). Measurement of intracellular superoxide levels revealed that exposure of cardiac cells to SI/R substantially increased the rate of oxidative stress (Fig. 1D). 64 µM KYNA treatment was shown to counteract this effect, significantly decreasing the cellular superoxide content compared to that observed in vehicle-treated cells (Fig. 1E). To further confirm that the most effective dose (i.e., 64 µM) improves the survival of cardiac cells undergoing SI/R in our experimental setup, LDH activity was measured from the supernatants of 64 µM KYNA/vehicle-treated cells as well as normoxic controls after SI/R, revealing that 64 µM KYNA treatment significantly diminished the SI/R-induced substantial increase in LDH activity (Fig. 1D). We did not find significant KYNA-derived effects on cellular viability, cell proliferation or migration under stress-free conditions (i.e., in the absence of SI/R) suggesting that the protective effect of KYNA observed in the settings of SI/R occurs due to direct cytoprotection rather than being the result of enhanced cell proliferation (Supplement. Figure 2).

### KYNA treatment attenuates apoptosis induced by SI/R

SI/R-induced cell death, at least in part, is due to apoptosis characterized by distinct cellular alterations including but not limited to cytoplasmic membrane blebbing, formation of apoptotic bodies, appearance of nuclear condensation and DNA double-strand breaks, as well as activation of caspases. Since KYNA treatment protected cardiomyoblasts against SI/R-triggered cell death, next we assessed whether KYNA alters the above-mentioned features of apoptosis. Light microscopy analysis of living cells revealed that KYNA significantly attenuated the SI/R-induced progression of plasma membrane blebbing (i.e., ameliorated the SI/R-induced increase in the ratio of cells showing morphology characteristic for irreversible apoptosis) and increased the ratio of cells exhibiting healthy morphology (Fig. 2A). Visualization of cell nuclei using DAPI staining revealed that the number of cells showing altered nuclear morphology due to apoptosis (i.e., appearance of either apoptotic bodies, micronuclei, DNA condensation or fragmentation) was significantly lower in the KYNA-treated group compared to that observed in the vehicle-treated cells (Fig. 2B-C). Furthermore, γ-H2AX immunostaining demonstrated that KYNA treatment seemed to reduce the ratio of cells showing moderate to high number of DNA double-strand breaks following SI/R (Fig. 2C-D). Western blot analysis confirmed the SI/R-induced increase of γ-H2AX, which was diminished significantly by KYNA treatment (Fig. 2E). In addition, SI/R was found to enhance the activity of caspase-3/-7 substantially, further corroborating its proapoptotic features; however, cells exposed to KYNA treatment showed an attenuated caspase activity (Fig. 2F). These findings revealed a modulatory effect of KYNA on apoptosis.

**Fig. 3:**
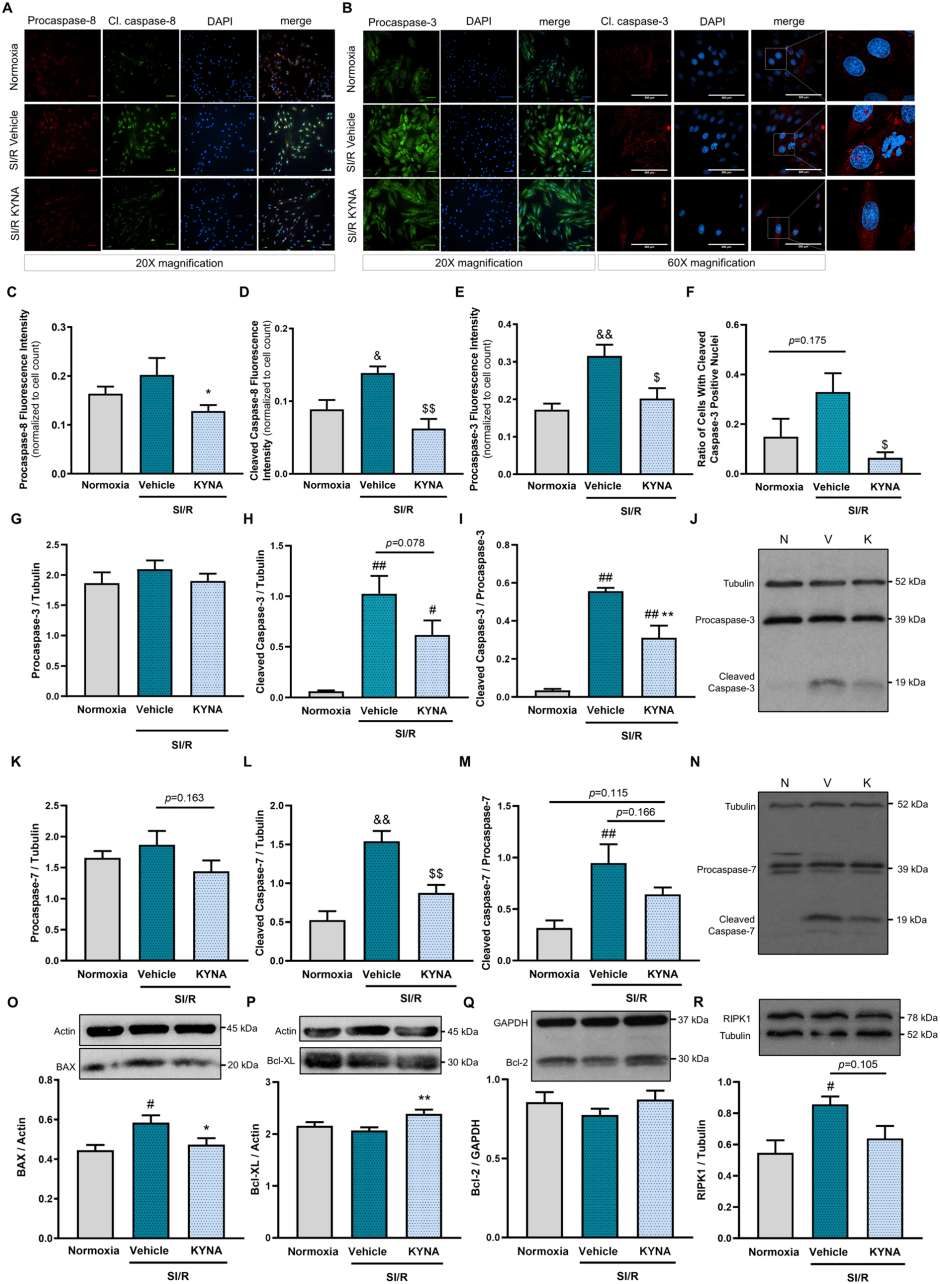
KYNA treatment reduces the level of SI/R-induced expression/activation of caspases and modulates the expression of apoptosis regulatory molecules beneficially. **A–B** Representative images from immunocytochemical investigations. **C–D** Levels of pro- and cleaved caspase-8 (*n* = 2 from 2 independent experiments, 5–6 randomly selected fields of view/sample). **E–F** Expression of procaspase-3 and ratio of cleaved caspase-3 positive cell nuclei (*n* = 2–3 from 2 independent experiments, 5–6 randomly selected fields of view/sample). **G–J** Protein level of both pro-/cleaved caspase-3 with representative pictures (N: normoxia, V: SI/R vehicle, K: SI/R KYNA). **K–N** Protein level of pro-/cleaved caspase-7 (N: normoxia, V: SI/R vehicle, K: SI/R KYNA). **O–R** Protein expression of BAX, Bcl-XL, Bcl-2 and RIPK1. For immunocytochemistry analysis, photos were taken at 20X magnification (scale bar: 500 µm) and fluorescence intensity was quantified using ImageJ software. For the analysis of nuclear cleaved caspase-3 expression, images were taken at 60X magnification (scale bar: 500 µm). Semiquantitative measurement of protein expression was performed via western blotting (caspases: *n* = 3–4/experiment, 2 independent experiments; modulatory proteins: *n* = 3–4/experiment, 2–3 independent experiments). Values were expressed as mean ± S.E.M., and were compared to normoxic or vehicle groups, ##*p* < 0.01 vs. Normoxia, #*p* < 0.05 vs. Normoxia, *p < 0.05 vs. SI/R + Vehicle, **p < 0.01 vs. SI/R + Vehicle, one-way ANOVA. Groups that did not show normal distribution were analyzed using Kruskal–Wallis test, $*p* < 0.05 vs. SI/R + Vehicle

To further verify the proposed antiapoptotic effect of KYNA, a proof-of-concept experiment was performed with a well-known apoptosis-inducer. Camptothecin, a topoisomerase-1 inhibitor [40], was applied at a concentration of 10 µM to induce apoptotic cell death, since this concentration had been shown to reduce cell viability substantially in our pilot experiments (Supplement. Figure 3A-B). KYNA treatment was shown to attenuate the camptothecin-induced cell death substantially (Fig. 2G, Supplement. Figure 3C-D), indicating that KYNA exerts a direct antiapoptotic effect. Therefore, next we focused on further examination of possible underlying antiapoptotic mechanisms in the SI/R setting.

### Apoptosis-related caspases are implicated in the KYNA-induced cardiocytoprotection

To reveal the potential caspase modulatory property of KYNA, the cellular expression of pro- and cleaved forms of apoptosis initiator caspase-8, as well as effector caspase-7 and -3 were determined. Immunostaining (Fig. 3A–F) demonstrated increased expression of the active cleaved form of caspase-8 due to SI/R that was significantly reduced by KYNA (Fig. 3A, D). The expression of the inactive precursor, procaspase-8 was not affected substantially by the treatment (Fig. 3A, C). However, the increased expression of procaspase-3 due to SI/R was considerably attenuated by KYNA as assessed by immunostaining (Fig. 3B, E). Similar effect was observed on the ratio of nuclei positive for cleaved caspase-3 (Fig. 3B, F). Western blot analysis showed significant increases in the cleaved forms of both caspase-3 (Fig. 3G–J) and -7 (Fig. 3 K–N) in response to SI/R, yet KYNA treatment seemed to attenuate or prevent these effects (Fig. 3G–N). The ratio of cleaved/procaspase-3 significantly decreased in KYNA-treated groups (Fig. 3I, J), while the decrease in the ratio of cleaved/procaspase-7 in the KYNA-treated group did not reach the level of statistical significance (Fig. 3M, N), most likely due to the reduced expression of procaspase-7 upon KYNA treatment (Fig. 3K, N). These findings suggest that KYNA attenuates SI/R-induced expression and/or activation of apoptosis-related caspases.

### KYNA influences key modulator proteins of apoptosis

We further examined whether pro- and antiapoptotic proteins, such as BAX, Bcl-XL, Bcl-2 or RIPK1 are implicated in the action of KYNA. Western blotting revealed a significant increase in the expression of proapoptotic BAX due to SI/R, however, KYNA was found to lower BAX levels significantly (Fig. 3O). KYNA treatment of cells undergoing SI/R increased the level of antiapoptotic Bcl-XL protein significantly compared to that observed in the vehicle-treated SI/R group (Fig. 3P). In case of Bcl-2, the expression exhibited a similar pattern to Bcl-XL, however, the differences did not reach the level of statistical significance (Fig. 3Q). Besides the Bcl-2 protein family members, RIP kinases may also regulate cell survival and death [41]. Figure 3R demonstrates that the expression of RIPK1 increased significantly upon SI/R, however, this SI/R-induced effect was not significant in the presence of KYNA treatment.

### The cytoprotective effect of KYNA is independent of the modulation of NMDA receptors

**Fig. 4:**
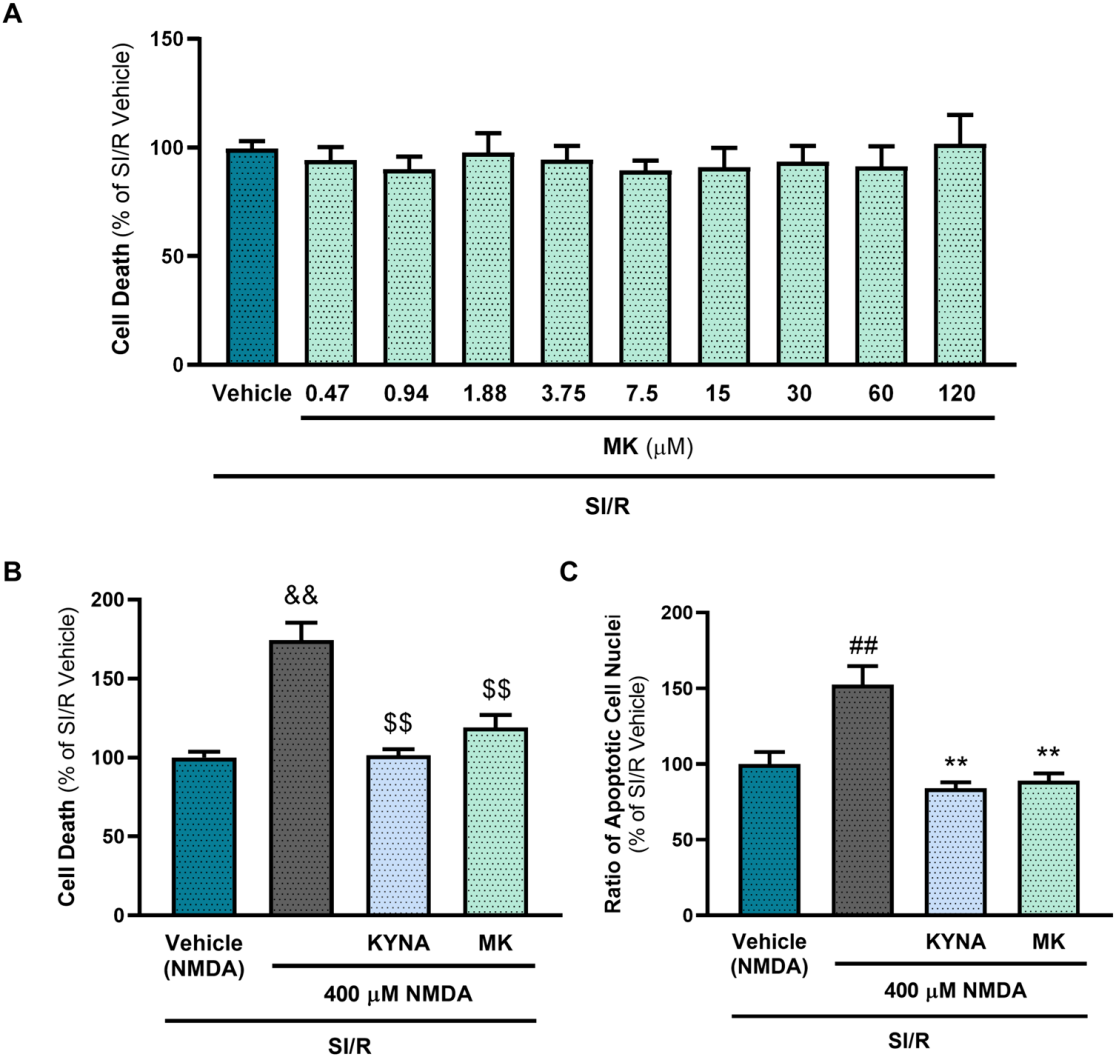
The cytoprotective effect of KYNA against SI/R-induced cell death does not seem to involve NMDA receptor mediated signaling. H9c2 cardiomyoblasts were exposed to SI/R while cells were treated with NMDA receptor antagonist MK-801 (0.47–120 µM), NMDA (400 µM), their vehicle or the combination of NMDA and KYNA (64 µM) or NMDA and MK-801 (7.5 µM), respectively. **A** The potential effect of MK-801 (0.47–120 µM) on SI/R-induced cell death was determined using calcein assay (*n* = 20–191, 11 separated experiments). **B** The effect of NMDA treatment and the combination of NMDA and KYNA or MK-801 against SI/R induced cell death was measured using calcein assay (*n* = 59–96 from 7 separated experiments). **C** Effects of NMDA or combined treatments (NMDA + KYNA or NMDA + MK-801) on apoptotic nuclear morphology was detected by DAPI staining (*n* = 2 from 2 independent experiments, 5 randomly selected fields of view/sample). Values were normalized to vehicle control group and were expressed as mean + S.E.M., **p < 0.01 vs. NMDA vehicle, ##*p* < 0.01 vs. NMDA, one-way ANOVA; &&*p* < 0.01 vs. NMDA vehicle, $$*p* < 0.01 vs. NMDA, Kruskal–Wallis test

Activation of NMDA receptors contributes to ischemic brain injury and its antagonism results in neuroprotection [42]. As KYNA is a well-known antagonist of NMDA receptors, one may speculate that KYNA protects cardiac cells against SI/R through the inhibition of NMDA receptor signaling. To address this hypothesis, cardiac cells exposed to SI/R were treated with MK-801, a potent, selective, non-competitive NMDA receptor antagonist. Surprisingly, the inhibition of NMDA receptors by MK-801 did not seem to attenuate the SI/R-induced cell death considerably in our experimental setup (Fig. 4A), suggesting that the activation of NMDA receptor is not involved in cardiac SI/R injury, and the cardiocytoprotection elicited by KYNA is independent of NMDA receptor antagonism. To demonstrate the presence of NMDA receptors in H9c2 cells and to confirm the antagonist effect of KYNA in our model, cells were treated with the receptor agonist NMDA with or without the known antagonists MK-801 or KYNA. Treatment with NMDA dose-dependently enhanced cell death (Supplement. Figure 4). Both antagonists, KYNA and MK-801 significantly protected H9c2 cells against NMDA-triggered cell death and apoptotic nuclear changes in SI/R (Fig. 4B-C). These results imply that the activation of NMDA receptors has no initiator and crucial role in the SI/R-induced apoptotic cell death of cardiomyocytes and KYNA may exert its cytoprotective effect via acting on other receptors.

### The cardiocytoprotective effect of KYNA involving antiapoptotic mechanisms is confirmed on primary cardiomyocytes

**Fig. 5:**
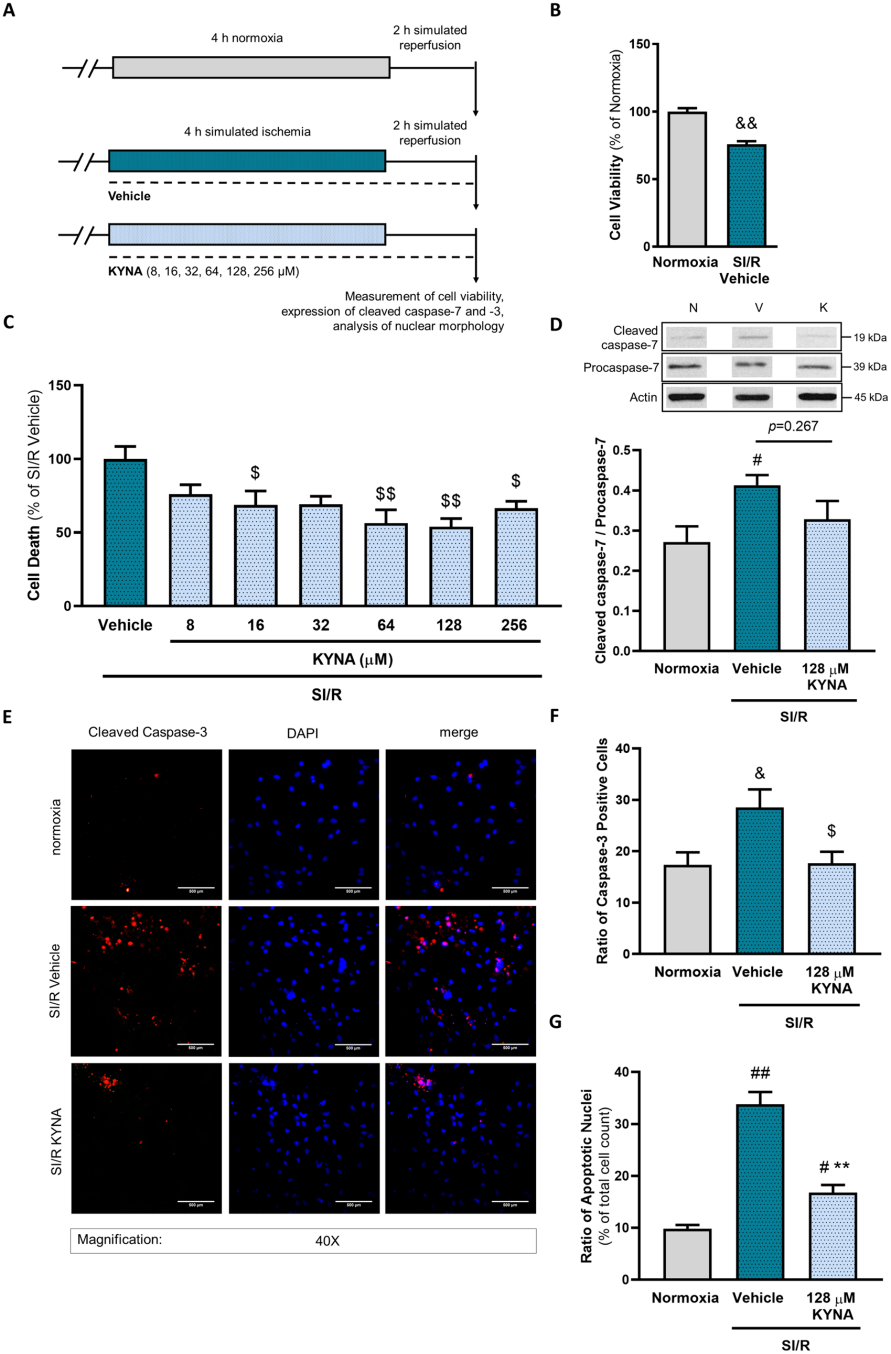
KYNA exhibits cardiocytoprotective effect against SI/R-induced cell death and caspase activation in primary neonatal rat cardiomyocytes. **A** Primary rat neonatal cardiomyocytes were exposed to 4 h simulated ischemia and 2 h simulated reperfusion. All cells were treated with KYNA or its vehicle (*n* = 7–14/experiment, 3 separated experiments). Cells of the control group were kept under normoxic conditions. **B–C** Calcein assay was used to determine the effect of SI/R on cell viability and the effect of KYNA treatment on SI/R-induced cell death. **D** The changes in the ratio of cleaved/procaspase-7 were examined using western blot analysis (*n* = 9 from 3 independent experiments). **E–F** The effect of KYNA on the ratio of caspase-3-positive cells exposed to SI/R was investigated using immunofluorescent staining (*n* = 3 from, 5 randomly selected fields of view/sample from 2 independent experiments). Scale: 500 µm. **G** Ratio of nuclei showing apoptotic morphology was measured using DAPI staining (*n* = 3–4, 5 randomly selected fields of view/sample from two independent experiments). Values were normalized to normoxic and vehicle control groups and were expressed as mean + S.E.M., &&*p* < 0.01 and &*p* < 0.05 vs. Normoxia, $*p* < 0.05 and $$*p* < 0.01 vs. SI/R Vehicle, Mann–Whitney or Kruskal–Wallis test; ##*p* < 0.01 and #*p* < 0.05 vs. Normoxia, **p < 0.01 vs. SI/R+Vehicle, one-way ANOVA

To confirm the relevance of our findings obtained on H9c2 cells, we tested whether KYNA protects primary cardiomyocytes against SI/R-induced cell death. Primary neonatal rat cardiomyocytes were subjected to SI/R with or without various doses of KYNA treatment as shown on Fig. 5A. Cell viability decreased significantly upon SI/R compared to normoxic controls (Fig. 5B). KYNA treatment dose-dependently attenuated the SI/R-induced cell death (Fig. 5C). The level of active, cleaved forms of caspase-3 and -7 were shown to increase in cells underwent SI/R as assessed by immunocytochemistry or western blotting (Fig. 5D–F). Nevertheless, the effect of SI/R on the activation of both caspase-3 and -7 in the presence of KYNA was not significant (Fig. 5D–F). We also examined the ratio of cell nuclei showing apoptotic morphology after DAPI staining and found that the substantial increase in the number of nuclei showing morphological hallmarks of apoptosis due to SI/R was significantly attenuated by KYNA (Fig. 5E, G). Our data obtained on primary cardiomyocytes confirm the cardiocytoprotective effect of KYNA that involves antiapoptotic effects.

## Discussion

Despite of basic and clinical research efforts, to date there is no effective therapy for myocardial I/R-induced cardiac cell damage and death. In the present study we confirmed the possibility to apply KYNA as a cytoprotective agent and investigated molecular and cellular mechanisms (including antiapoptotic effects and NMDA receptor signaling) potentially involved in the cardiocytoprotective effect of KYNA against I/R-induced cellular demise.

We found that exogenously administered KYNA, that is proved to be tolerated by cells in a broad concentration range under stress-free conditions [43, 44], could dose-dependently protect H9c2 cells and neonatal cardiomyocytes from SI/R-induced cell death and oxidative stress. This is in line with observations of Olenchock et al*.*, who showed that exogenously administered KYNA can decrease the infarct size significantly in mice subjected to coronary ligation and reperfusion [28].

The mechanisms contributing to I/R injury are multifactorial and comprise both cell death and survival processes [2, 3]. Besides necrosis, apoptosis is an important contributor to I/R-induced cell death as well and may serve as a potential target for cardioprotection [2, 4, 45]. Apoptosis can be defined by characteristic morphological changes such as cell shrinkage, dynamic membrane blebbing [46], alterations in nuclear morphology (*e.g.*, DNA condensation and fragmentation, formation of micronuclei and DNA breaks, as well as appearance of apoptotic bodies) [35]. Here we provided several lines of evidence suggesting that these characteristic apoptosis-related alterations induced by SI/R can be delayed, reduced, or even reverted if cells (either H9c2 cardiomyoblasts or primary cardiomyocytes) receive KYNA treatment during SI/R. We demonstrated for the first time that KYNA treatment of cardiac cells was able to reduce the number of nuclei showing apoptotic morphology, the frequency of DNA double-strand breaks and had beneficial effects on cell membrane blebbing.

Activation of the caspase cascade is a fundamental episode in apoptosis [35, 47]. In the present study, SI/R was shown to increase the activity of caspase-8, -3 and -7. Importantly, KYNA treatment either attenuated or significantly diminished these SI/R-induced changes. Based on these results we propose that the modulation of caspase activation is involved in the KYNA-associated cytoprotection against SI/R. This modulatory feature of KYNA on caspase activation is further supported by findings demonstrating that KYNA withdrawal elicits an increase in neuronal apoptosis in an in vitro model of epilepsy [48]. Furthermore, pre-treatment of SH-SY5Y and SK-N-SH cells with KYNA was shown to reduce the 1-methyl-4-phenylpyridinium-induced neuronal cell death through the attenuation of caspase-9/-3 activities in an in vitro model of Parkinson’s disease [49].

**Fig. 6:**
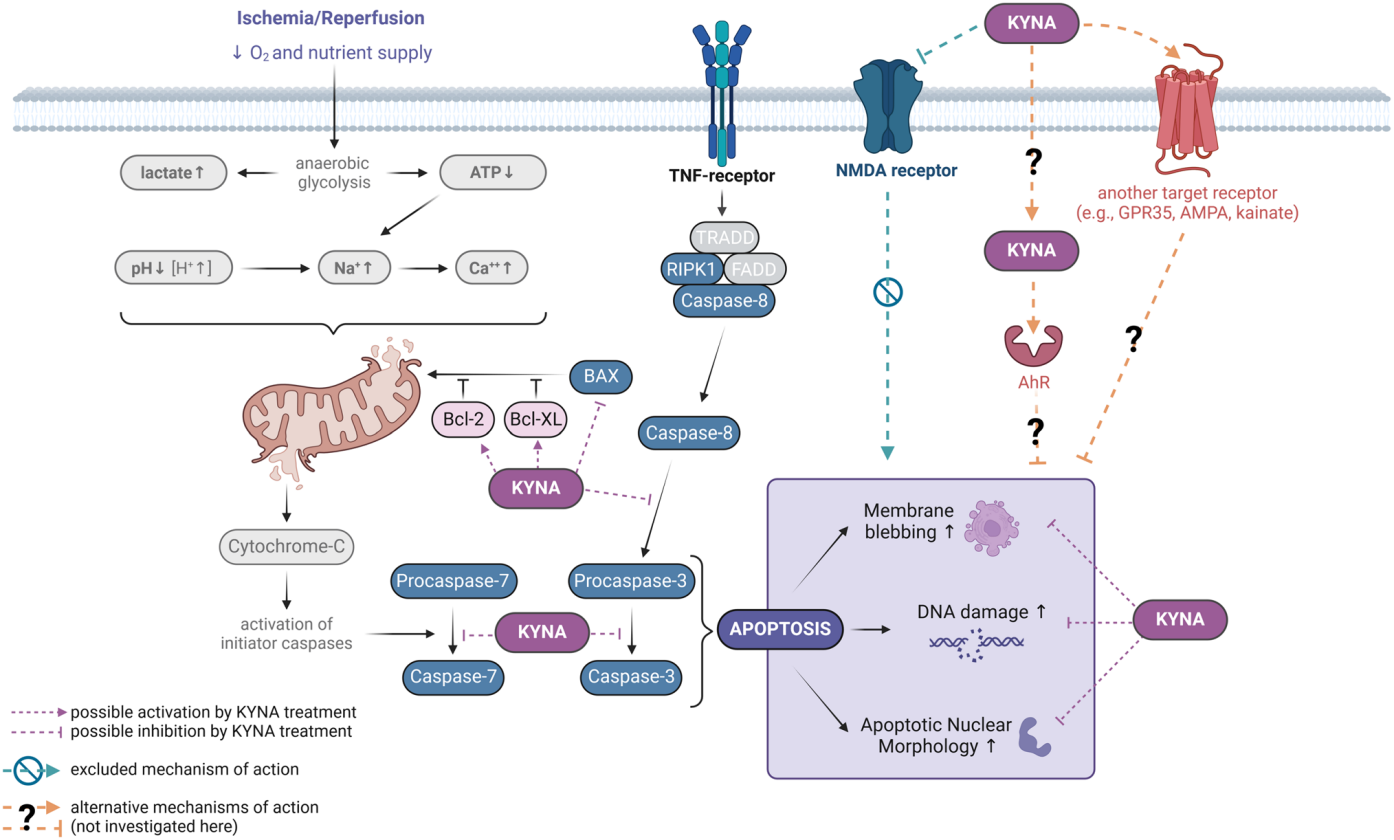
The proposed mechanisms involved in the cytoprotective effect of KYNA against SI/R-induced cardiac cell demise. *AMPA* α-amino-3-hydroxy-5-methyl-4-isoxazolepropionic acid, *AhR* aryl hydrocarbon receptor, *ATP* adenosine triphosphate, *BAX* Bcl-2 associated X, *Bcl-2* B-cell lymphoma 2, *Bcl-XL* B-cell lymphoma extra large, *KYNA* kynurenic acid, *GPR35* G protein-coupled receptor 35, *NMDA* N-methyl-D-Aspartate, *TNF* tumor necrosis factor. The figure was created using BioRender.com

Apart from the caspase cascade, the expression of additional pro- and antiapoptotic modulator proteins was also affected by KYNA during SI/R. BAX, a core proapoptotic regulator of the intrinsic apoptotic pathway, is activated and oligomerized at the mitochondrial outer membrane to mediate its permeabilization upon harmful stimuli, which is considered as a key step of apoptosis initiation [50]. In our study, SI/R induced a substantial increase in the expression of BAX, which was reverted by KYNA treatment. A similar feature was observed previously by Lee et al*.*, who proposed that KYNA exerts neuroprotection in an in vitro model of Parkinson’s disease at least in part due to the down-regulation of BAX expression [49]. Besides BAX, Bcl-2 and Bcl-XL also play important roles in the regulation of mitochondria-dependent cell death pathways [51]. These antiapoptotic proteins counteract the pore-forming activity of BAX via direct inhibitory interactions [51, 52]. Furthermore, Bcl-XL has been shown to modulate mitochondrial ATP synthesis, autophagy and mitosis [53]. In the present study, KYNA-treatment was shown to increase the level of Bcl-XL protein significantly compared to that observed in vehicle-treated cells underwent SI/R. The expression of Bcl-2 protein exhibited a similar, but less pronounced tendency without significant change. We also demonstrated that SI/R increased the protein expression of RIPK1 considerably, however, this effect was not significant compared to normoxic controls when KYNA treatment was applied. RIPK1-containing complexes are also involved in the regulation of apoptosis as overexpression of RIPK1 has been described to induce death receptor-mediated apoptosis [54, 55]. These results suggest that KYNA treatment beneficially affects both proapoptotic (BAX, RIPK1) and antiapoptotic (Bcl-XL, Bcl-2) cellular processes (Fig. 6).

Receptor-mediated modulation of downstream signaling pathways is most likely the mechanism laying behind the KYNA-induced cytoprotection. KYNA binds to several receptors (Supplementary Table 1) exerting antagonistic effects on ionotropic glutamate receptors, such as NMDA, AMPA and kainate receptors, while eliciting agonistic properties on AhR and GPR35 receptors [15]. It has been demonstrated that KYNA exerts its neuroprotective effect at least in part by controlling the excessive activation of NMDA receptors, thereby preventing excitotoxic cellular damage [56, 57]. It is also known that NMDA receptors are not exclusively expressed in the central nervous system, but are also present in peripheral organs, including the heart, lungs, kidneys, and pancreas [58]. As NMDA receptor activation has been shown to substantially contribute to ischemia/reperfusion-induced damage of the nervous tissue [59], as well as to stimulate hypoxia-induced apoptosis in neurons [60], we examined the possible involvement of NMDA receptor antagonism in the cardioprotective effect of KYNA. According to our results, NMDA receptor activation by NMDA treatment during SI/R caused further increase of cell death. KYNA and another potent NMDA receptor antagonist, MK-801, were able to ameliorate this additional effect, demonstrating the expression and functionality of NMDA receptors in the tested cells. However, in comparison to KYNA, MK-801 treatment alone did not attenuate SI/R-induced cell death significantly, suggesting that NMDA receptor antagonism is not the main mechanism in the protective effect of KYNA treatment in our experimental model (Fig. 6.). In fact, other target receptors may mediate the KYNA-derived protection in cardiac cells. This hypothesis is supported by the findings of Wyant et al*.* suggesting that GPR35 receptor agonism contributes to the protective effect of KYNA in cardiac I/R injury [29].

Taken together, our data demonstrates the involvement of apoptosis in the cellular events triggered by SI/R and suggest that the cardiocytoprotective effect of KYNA engages alleviation of several cellular and morphological changes related to apoptosis induced by SI/R, independently of NMDA receptor antagonism.

### Limitations of the study

Although we have provided several pieces of evidence to prove the cardiocytoprotective effect of KYNA and to reveal the underlying mechanism of action, this work is not without limitations. Firstly, we obtained results from in vitro experiments using cardiomyoblast and cardiomyocyte cultures. Therefore, our study is limited to demonstrate some aspects of the KYNA-derived cytoprotection that requires the presence of other cell types. Nevertheless, some of our findings have been confirmed by others using ex vivo and in vivo experimental setups. Moreover, the already proven immunomodulatory properties of KYNA might contribute further to potential beneficial effects under in vivo circumstances. Secondly, we have excluded the involvement of NMDA receptor antagonism in the KYNA-induced cytoprotection, while others implicated the importance of GPR35 agonism. However, further studies are needed to identify whether other KYNA target receptors (e.g., AMPA, kainate receptors, nicotinic acetylcholine receptor, aryl-hydrocarbon receptors) are involved in the protection elicited by KYNA. Lastly, our study focused on apoptosis primarily, however, potential involvement of other types of regulated cell death events, such as autophagy, necroptosis, ferroptosis or pyroptosis might be also relevant and remains to be investigated. Despite these limitations, we have provided novel details on the mechanism of action of KYNA on cardiac cells and identified the antiapoptotic aspects of the KYNA-induced protection on the cellular level. Nevertheless, future studies are needed to clarify further the molecular actions of KYNA against I/R injury.

## Conclusions

We have shown here a variety of different approaches (i.e., apoptosis related morphological changes, DNA damage, caspase activation, and apoptotic signaling) to prove that KYNA exerts a clear antiapoptotic effect which contributes to the cardiocytoprotective effect of KYNA against SI/R injury. Our study is the first to implicate antiapoptotic mechanisms in the cytoprotective effect of KYNA in cardiac cells. However, NMDA receptor signaling does not seem to play a major role in the beneficial effects of KYNA.

## Supplementary Information

Below is the link to the electronic supplementary material.

Supplementary file1 (DOCX 265 KB)

Supplementary file2 (DOCX 17 KB)

## Data Availability

All data supporting the findings of this study are available from the corresponding author on a reasonable request.
