# Supplementary material for: Kynurenic acid protects against ischemia/reperfusion injury by modulating apoptosis in cardiomyocytes
Source: Apoptosis. 2024 Aug 17;29(9-10):1483–98. doi: 10.1007/s10495-024-02004-w (PMC11416393; doi:10.1007/s10495-024-02004-w)
Supplement: Supplementary file 1 — Supplementary file1 (DOCX 265 KB) [file 10495_2024_2004_MOESM1_ESM.docx]

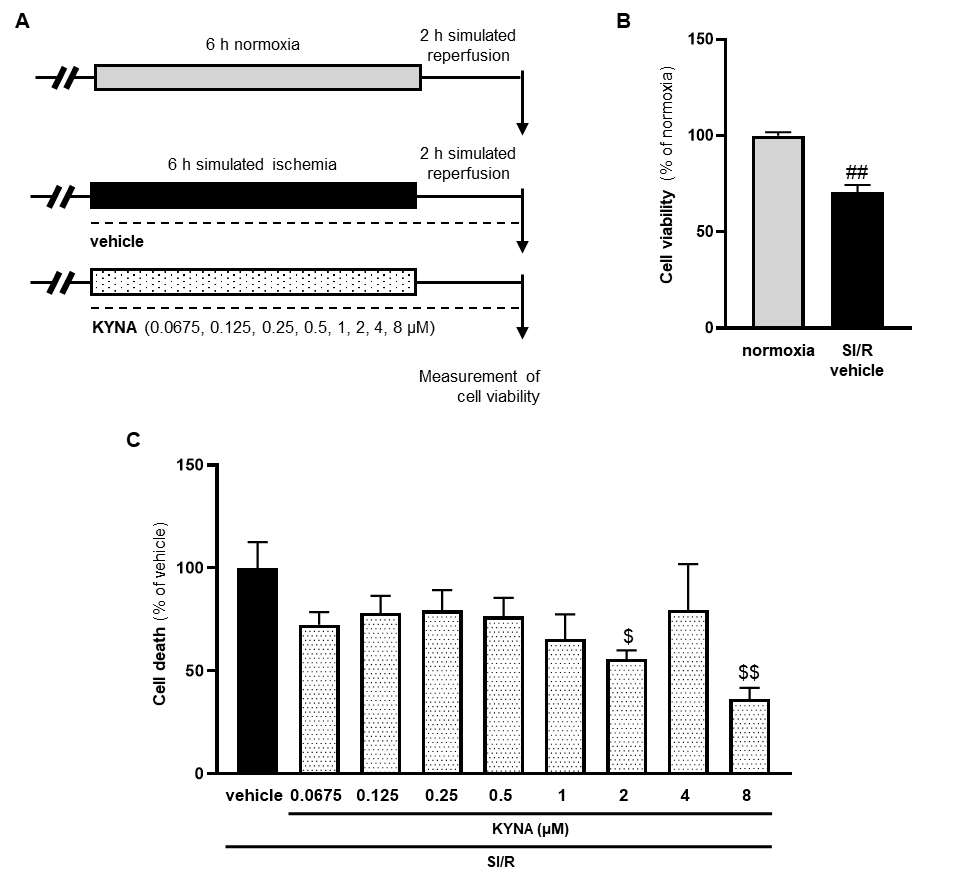


**Supplementary Figure 1. The effect of kynurenic acid (KYNA) in the nM to low μM concentration range on simulated ischemia/reperfusion (SI/R)-induced damage of H9c2 cells.** (**A**) H9c2 cardiomyoblasts were subjected to SI/R protocol (i.e., 6 h simulated ischemia followed by 2 h simulated reperfusion) while the cells were treated with KYNA (nM to low μM concentration range) or its vehicle (treated cells: n=7-14, controls: n=14-21, from two independent experiments). Cells of the control group were kept under normoxic conditions. (**B-C**) Calcein assay was performed to identify the effect of SI/R on cell viability and the impact of KYNA treatment on SI/R-induced cell death. Values were normalized to normoxic (**B**) or vehicle (**C**) control groups and were expressed as mean + S.E.M., *##p* < 0.01 vs. normoxia, student’s T-test. The non-normal distribution showing groups were analyzed using Kruskal-Wallis test, *$p*<0.05 vs. SI/R + vehicle, *$$p* < 0.01 vs. SI/R + vehicle.

**
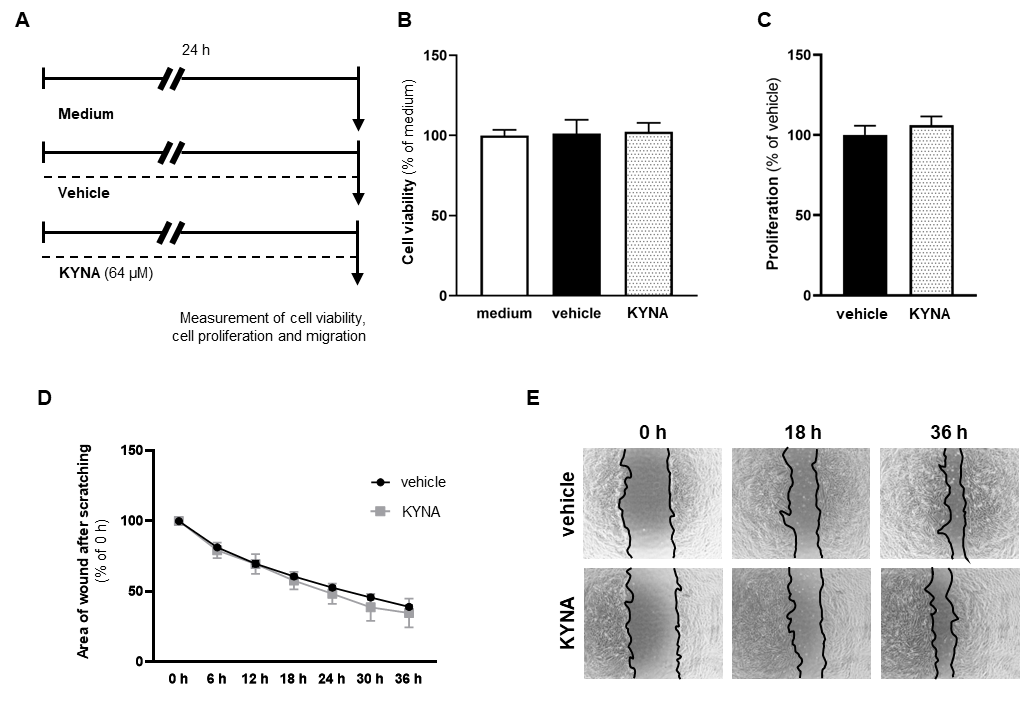
**

**Supplement Figure 2. Kynurenic acid (KYNA) has no significant effects on H9c2 cells under normoxic conditions.** (**A**) H9c2 cardiomyoblasts were treated with 64 μM KYNA under normoxic conditions for 24 h. (**B**) Calcein assay was performed to measure the cell viability (n=7-14/experiment, 3 separated experiments). (**C**) To investigate whether KYNA influences cell proliferation under stressfree, normoxic conditions, BrdU assay was performed using a Cell proliferation ELISA kit (Roche® Life Science Products, Cat# 11647229001) according to the manufacturer’s instructions. Briefly, to quantity cell proliferation based on incorporation of BrDU into the newly synthesized DNA, H9c2 cells were plated into 96-well plates and were pre-treated with 64 µM KYNA or its vehicle for 24 h followed by 20 h incubation with 10 μM BrdU-labeling solution. Cells were then fixed (FixDenat solution, 30 min) and treated with horseradish peroxidase conjugated BrdU antibody (90 min, RT). Cells were then incubated with tetramethylbenzidine substrate for 30 min, followed by measurement of absorbance at 450/620 nm using a microplate reader. Data were normalized to the corresponding vehicle-treated control group. (**D-E**) Wound-healing assays was performed to investigate whether KYNA can influence cell proliferation and migration (n=6-8/groups, 3 independent experiments). For this, H9c2 cells were seeded into 24-well plates at a density of 5 x 10^4^, covered with growth medium and maintained for 2 days to reach a suitable confluence. A wound was scratched through the center of each well using 200 µL pipette tips. The supernatant was then changed to growth medium supplemented with 64 µM kynurenic acid or its vehicle after scratching. A random field of cell-free zone and wound edges was selected, marked, and photographed 0, 6, 12, 18, 24, 30 and 36 hours after scratching in case of each well. Images were analyzed using ImageJ software, the area of cell-free zones was measured and compared to vehicle-treated groups. Values were expressed as mean + S.E.M. and compared to corresponding medium or vehicle groups. No significant effects of KYNA were detected on cellular viability or proliferation under normoxic condition.

**
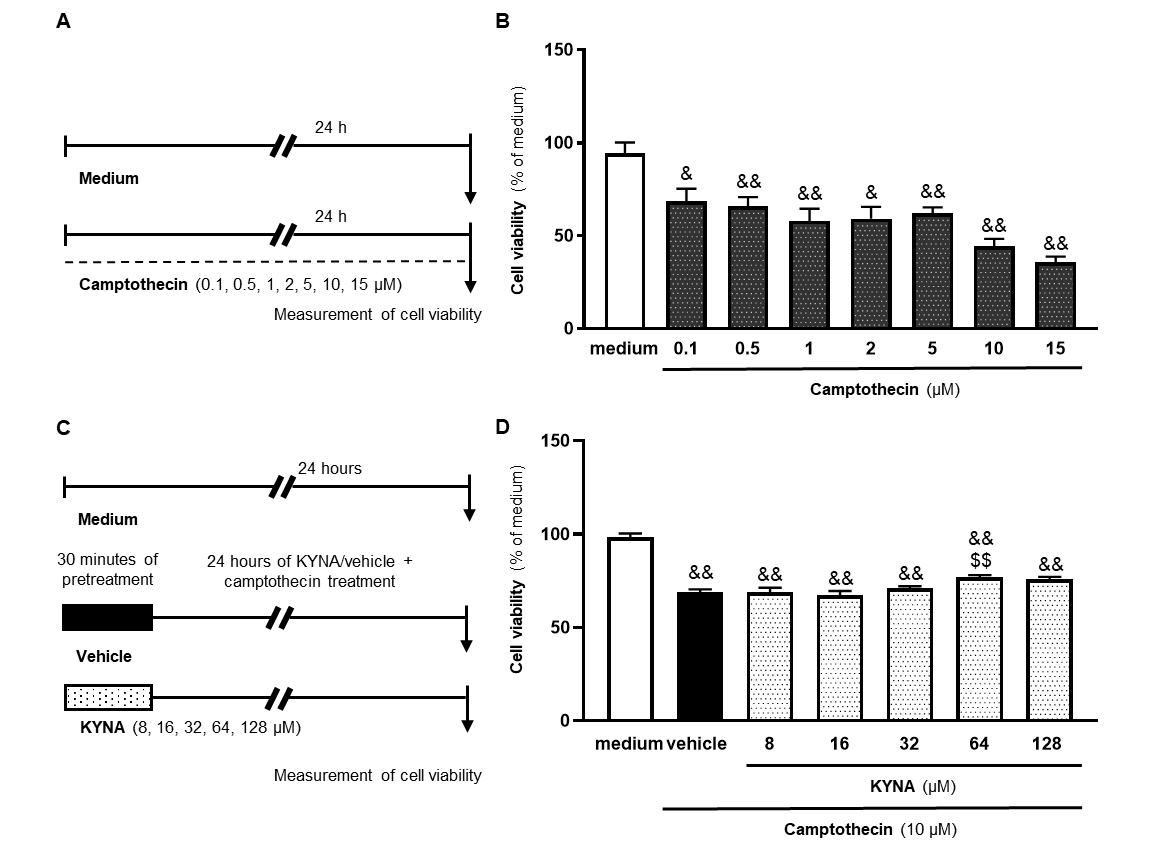
**

**Supplement Figure 3. Camptothecin-induced cell death and its modulation by kynurenic acid (KYNA)**. (**A-B**) H9c2 cardiomyoblasts were exposed to 0.5-15 µM camptothecin for 24 h to induce apoptosis and MTT assay was used to assess the optimal camptothecin concentration. (**B**) 10 µM camptothecin treatment was chosen for testing the effect of KYNA on camptothecin-induced cell death. (**C-D**) Cells were exposed to camptothecin treatment with or without KYNA for 24 h, followed by the measurement of cell viability using MTT assay (n=6-12/experiment, from 3 separated experiments). Values were expressed as mean ± S.E.M. and compared to medium or vehicle groups. Groups showed non-normal distribution and were analyzed using Kruskal-Wallis test, *&p < 0.05 vs. medium, &&p < 0.01 vs. medium, $$p < 0.01 vs. vehicle*.

**
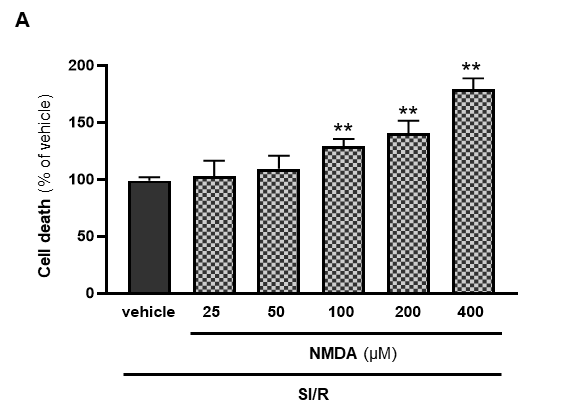
**

**Supplementary Figure 4. Determination of the effect of N-methyl-D-aspartate (NMDA) on simulated ischemia/reperfusion (SI/R)-induced cell death.** H9c2 cardiomyoblasts were exposed to SI/R (as was described above) while cells were treated with 25-400 µM NMDA. A group of cells was kept under normoxic conditions (treated cells: n=7-14, controls: n=14-28/experiment, from 10 independent experiments). Cell viability was detected using calcein assay and cell death was calculated from the viability data. Values were normalized to NMDA vehicle control groups and expressed as mean + S.E.M., ***p <* 0.01 vs. NMDA vehicle, one-way ANOVA.
