# Supplementary material for: Kynurenic acid protects against ischemia/reperfusion injury by modulating apoptosis in cardiomyocytes
Source: Apoptosis. 2024 Aug 17;29(9-10):1483–98. doi: 10.1007/s10495-024-02004-w (PMC11416393; doi:10.1007/s10495-024-02004-w)
Supplement: Supplementary file 2 — Supplementary file2 (DOCX 17 KB) [file 10495_2024_2004_MOESM2_ESM.docx]

| **Target receptors** | | **Effect of KYNA** |
| --- | --- | --- |
| **NMDA receptor** | ionotropic glutamate receptor | **antagonist** |
| **AMPA receptor** | ionotropic glutamate receptor | **antagonist** |
| **kainate receptor** | ionotropic glutamate receptor | **antagonist** |
| **α7nACh receptor** | nicotinic ACh receptor | **antagonist** |
| **Aryl hydrocarbon receptor** | ligand-activated transcription factor | **agonist** |
| **GPR35 receptor** | G-protein coupled receptor | **agonist** |

**Supplementary Table 1.** **Receptors Targeted by KYNA
Abbreviations**: NMDA: N-methyl-D-aspartate; AMPA: α-Amino-3-hydroxy-5-methyl-4-isoxazolepropionic acid; α7nACh: α7 nicotinic acetylcholine receptor; GPR35: G-protein-coupled receptor 35

| **Antibody** | **Source** | **Identifier** |
| --- | --- | --- |
| Rabbit polyclonal anti-α-Tubulin | CST | Cat#2144 (RRID: AB_2210548) |
| Rabbit monoclonal anti-β-Actin | CST | Cat#4970 (RRID: AB_2223172) |
| Rabbit monoclonal Anti-Phospho-γ-H2AX (Ser139) | Thermo Fisher Scientific | Cat#MA5-33062 (RRID: AB_2810155) |
| Rabbit monoclonal anti-BAX | CST | Cat#14796 (RRID: AB_2716251) |
| Rabbit polyclonal anti-Bcl-2 | Abcam | Cat#ab196495 (RRID: AB_2924862) |
| Rabbit monoclonal anti-Bcl-XL | CST | Cat#2764 (RRID: AB_2228008) |
| Rabbit monoclonal anti-GAPDH | CST | Cat#2118 (RRID: AB_561053) |
| Rabbit monoclonal anti-Caspase-8 | CST | Cat#4790 (RRID: AB_10545768) |
| Mouse monoclonal anti-Cleaved Caspase-8 (Asp384) | CST | Cat#9748 (RRID: AB_331181) |
| Rabbit monoclonal anti-Caspase-3 | CST | Cat#14220 (RRID: AB_2798429) |
| Rabbit monoclonal anti-Cleaved Caspase-3 (Asp175) | CST | Cat#9664 (RRID: AB_2070042) |
| Rabbit polyclonal anti-Caspase-7 | CST | Cat#9492 (RRID: AB_2228313) |
| Rabbit monoclonal anti-RIP | CST | Cat#3493 (RRID: AB_2305314) |
| Anti-rabbit IgG (H+L), F(ab')2 Fragment (Alexa Fluor® 488 Conjugate) | CST | Cat#4412 (RRID: AB_1904025) |
| Anti-mouse IgG (H+L), F(ab')2 Fragment (Alexa Fluor® 488 Conjugate) | CST | Cat#4408 (RRID:AB_10694704) |
| Anti-rabbit IgG (H+L), F(ab')2 Fragment (Alexa Fluor® 647 Conjugate) #4414 | CST | Cat#4414 (RRID: AB_10693544) |
| Goat Anti-Rabbit Immunoglobulins/HRP | Agilent | Cat#P0448 (RRID: AB_2617138) |

**Supplementary Table 2.** **Primary and Secondary Antibodies Used for Immunocytochemistry and Western Blotting**
